# A Case of Incomplete Scimitar Syndrome in a Quinquagenarian Male

**DOI:** 10.7759/cureus.78315

**Published:** 2025-01-31

**Authors:** Hudson P Franca, Priscila Sole, Sahar S Abdelmoneim, Matthew Razavian, Sabas Gomez

**Affiliations:** 1 Internal Medicine, Larkin Community Hospital - Palm Springs Campus, Hialeah, USA; 2 Medicine, Centro Universitário Lusíada, São Paulo, BRA; 3 General Internal Medicine, Larkin Community Hospital - Palm Springs Campus, Hialeah, USA; 4 General Internal Medicine/Cardiovascular Medicine, Assiut University Heart Hospital, Assiut, EGY; 5 Cardiology, Larkin Community Hospital - South Miami, South Miami, USA

**Keywords:** adult congenital heart disease, anomalous pulmonary vein, congenital cardiovascular anomaly, image findings, scimitar syndrome

## Abstract

Scimitar syndrome is a rare congenital cardiopulmonary condition characterized by an anomalous pulmonary venous return from the right lung to the inferior vena cava (IVC). Most patients are diagnosed during infancy. Incomplete scimitar syndrome refers to a variant, where only a portion of the right lung drains abnormally into the IVC without associated cardiac defects. This case involves a 59-year-old man who presented with severe dizziness and hypertensive emergency with a blood pressure of 234/122 mmHg. His past medical history includes hypertension, cerebrovascular accident, prior myocardial infarction, and diabetes mellitus. A computed tomography (CT) without contrast demonstrated an aberrant connection of the right lower pulmonary vein to the IVC. The patient did not have associated congenital heart defects (incomplete scimitar syndrome). He was conservatively treated for his comorbidities with clinical improvement. Incomplete scimitar syndrome manifests late in adulthood as dyspnea during exertion secondary to the development of pulmonary hypertension. Hence, the cornerstone of treatment for pulmonary hypertension is medical control. By concentrating on the adult appearance of incomplete scimitar syndrome, long-term treatment and care may result from the identification of more individuals with incomplete scimitar syndrome as imaging methods advance and awareness increases. Future longitudinal outcome research for such rare conditions is crucial to fill up these information gaps.

## Introduction

Total anomalous pulmonary venous return (TAPVR) is a critical congenital heart defect (CHD), with an incidence of one in every 7.809 live births in the United States, frequently associated with atrial septal defect. In these cases, surgical intervention is required after birth [1,2]. Another form of anomalous venous connection is the partial anomalous pulmonary venous return (PAPVR), where one or more, but not all the pulmonary veins abnormally drain into the right atrium, directly or indirectly, with associated CHD. TAPVR and PAPVR are uncommon congenital heart anomalies. They represent less than 1% of all congenital heart diseases [3]. Scimitar syndrome is a rare congenital cardiopulmonary disorder. It is a specific form of PAPVR, occurring in two out of every 100,000 live births. It accounts for 3-5% of all PAPVR cases and involves an abnormal pulmonary vein draining the right lung into the inferior vena cava (IVC), leading to a left-to-right shunt. The clinical presentation of scimitar syndrome spans a wide spectrum, ranging from severe symptoms in infancy to milder or even asymptomatic cases in adulthood, suggesting that the condition may be underdiagnosed in the adult population; thus, the incidence may be higher in the adult [3,4].

Diagnosis is through imaging techniques, including echocardiography, computed tomography (CT), magnetic resonance imaging (MRI), and CT angiography. The majority of cases of scimitar syndrome are diagnosed in childhood, and treatment often involves surgical intervention, which generally has favorable outcomes. However, long-term follow-up is essential to monitor for potential complications such as arrhythmias, pulmonary hypertension, or recurrence of symptoms [5,6]. Atrial tachyarrhythmias, including atrial fibrillation (AF) and atrial flutter, have been reported in patients with incomplete scimitar syndrome as a sequela of structural cardiac abnormalities, such as left atrial enlargement and pulmonary hypertension [7].

The distinguishing feature between an incomplete scimitar and a complete scimitar is mainly based on the degree of venous anomaly and associated congenital structural heart features. In the incomplete scimitar, the anomalous pulmonary venous drainage is incomplete, and patients may not present with obvious or severe respiratory symptoms early in life and subtle findings on imaging, hence the late adulthood incidental diagnosis. Thus, the incomplete scimitar syndrome, because of the milder form in adult patients, is rarely documented in the literature [5].

This report discusses a 59-year-old man diagnosed with incomplete scimitar syndrome. The case adds to the growing knowledge of this rare condition, especially in the adult population. It emphasizes the necessity of raising awareness of the incomplete variant, which frequently goes undetected because of its modest appearance. We also review related literature on similar cases, offering valuable insight to support clinical decision management. The patient was informed that data concerning the case would be submitted for publication, and he provided informed consent.

## Case presentation

A 59-year-old male presented to the emergency department with a chief complaint of dizziness and nausea for three days. Past medical history included hypertension, diabetes mellitus, prior myocardial infarction, and cerebrovascular accident. The patient denied vertigo, tinnitus, fever, chills, chest pain, dyspnea, palpitations, abdominal pain, slurring of speech, or syncope episodes. He denied any history of recurrent respiratory infections. His home medications included aspirin, clopidogrel, clonidine, furosemide, spironolactone, metoprolol, metformin, and insulin. In the emergency department (ED), the patient was alert and oriented to time, person, and place, and vital signs were recorded as blood pressure 234/122 mmHg, regular rhythm with a heart rate of 61 beats per minute (bpm), temperature of 99 F, respiratory rate of 20/min, and saturation at 98% on room air. Physical examination revealed a systolic pulmonary outflow murmur associated with a widely split and fixed second heart sound in the left parasternal area. There was no jugular venous distention. Neurological exam revealed mild weakness of the left side, due to previous cerebrovascular accident sequela, but was otherwise unremarkable.

He was initially treated for a hypertensive emergency. He received 10 mg of hydralazine, which decreased the blood pressure to 191/90 mmHg, followed by labetalol 10 mg, which decreased it further to 164/92 mmHg. Initial laboratory testing in the ED (Table 1) revealed normal hemoglobin at 13.2 g/dL; elevated leucocytes at 13,400/uL, neutrophils at 90.3%, and platelet count at 648,000/uL (normal range: Na, 135 mEq/L; K, 3.7 mEq/L; Ca, 9.5 mg/dL).

**Table 1:** Complete laboratory results

Laboratory	Results	Reference range
Complete blood count		
Hemoglobin, blood	13.2 g/dL	14-18 g/dL
Hematocrit, blood	40.3%	42-50%
Mean Corpuscular Volume	85 fL	80-98 fL
Leucocyte count	13,400/uL	4,500-11,000/uL
Neutrophils	90.3%	50-70%
Platelet count	648,000/uL	150,000-450,000/uL
Reticulocyte	4.75%	0.5%-1.5%
Complete metabolic panel		
Sodium, serum	135 mEq/L	136-145 mEq/L
Potassium, serum	3.7 mEq/L	3.5-5.0 mEq/L
Urea nitrogen, blood	29 mg/dL	8-20 mg/dL
Creatinine, serum	1.0 mg/dL	0.7-1.3 mg/dL
Anion gap, serum	14 mmol/L	7-13 mmol/L
Calcium, serum	9.5 mg/dL	8.6-10.2 mg/dL
Urinalysis and urine microscopy		
Color	Brown	Yellow/Straw
Clarity	Cloudy	Clear
Glucose, urine	500	Negative
Ketone	15	Negative
Gravity	>=1.030	1.005-1.025
PH, urine	6.0	4.5-8
Protein, urine	100 mg/dL	<100 mg/24 h
Nitrite	Negative	Negative
Red blood cells, urine	20-40/hpf	<= 3 red blood cells/hpf
Leukocyte esterase	Small	Negative
White blood cells, urine	40-80/hpf	<= 2 white blood cells/hpf
Bacteria, urine	Moderate	Negative

**Figure 1:**
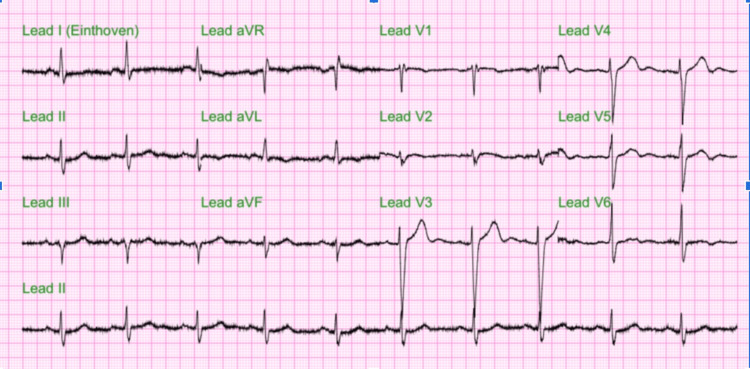
Electrocardiogram (EKG) showed a sinus rhythm of 90 bpm with no significant ST segment T wave changes

**Figure 2:**
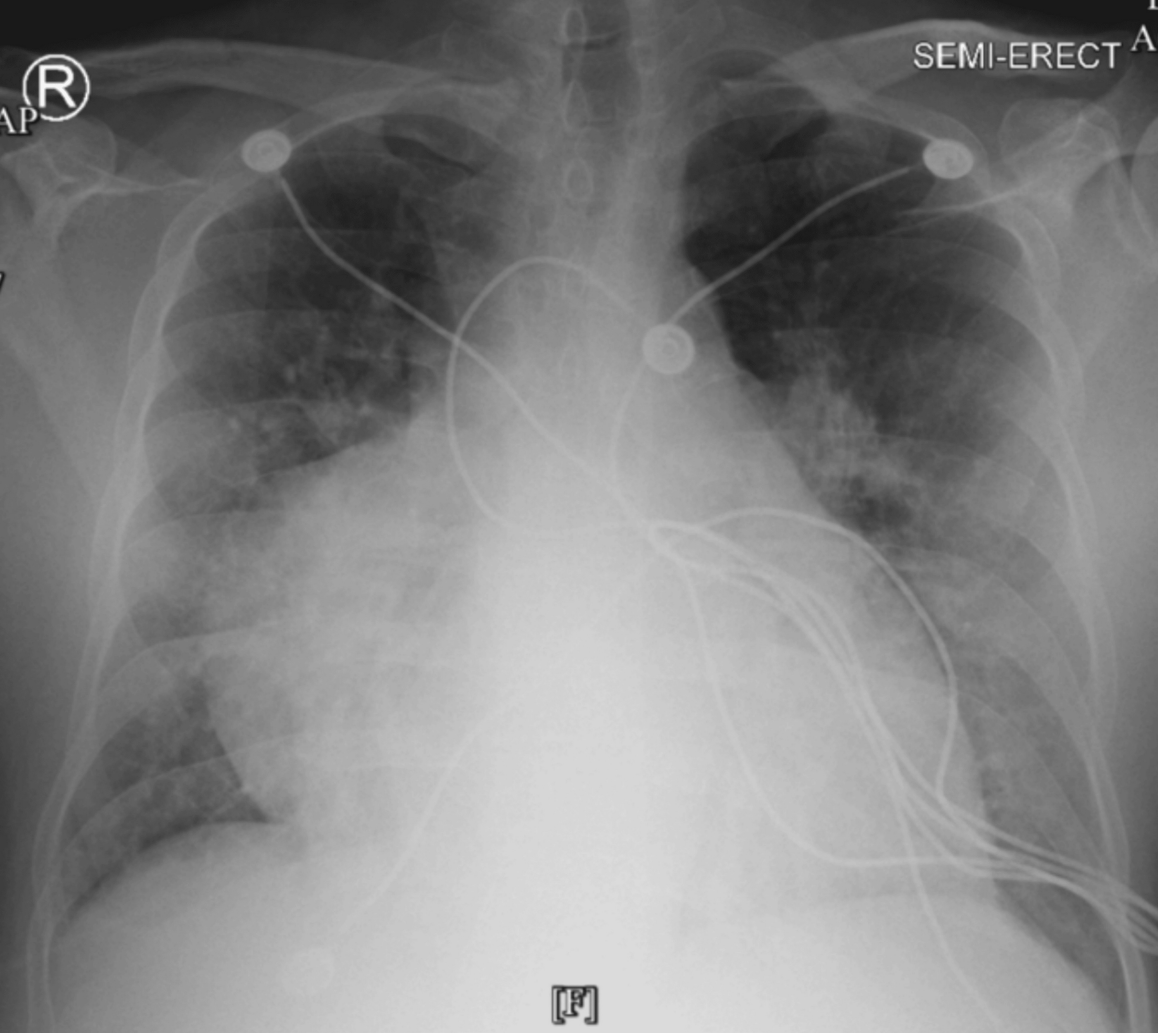
Admission chest radiograph demonstrating prominent vascularity bilaterally

**Figure 3:**
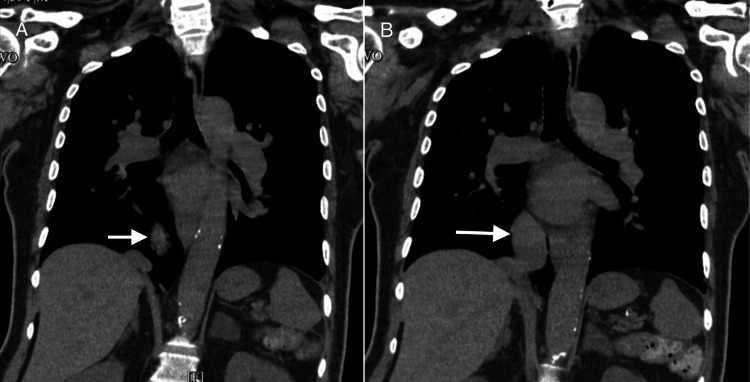
Chest computed tomography (CT) without contrast (coronal reconstruction at posterior level in A and more anteriorly in image B), demonstrating the scimitar vein, the aberrant connection of the pulmonary vein into the Inferior vena cava Chest CT at the level of the mid-to-lower thorax, capturing the mediastinal structures, including the lower portion of the trachea, bronchi, and adjacent lung parenchyma.

**Figure 4:**
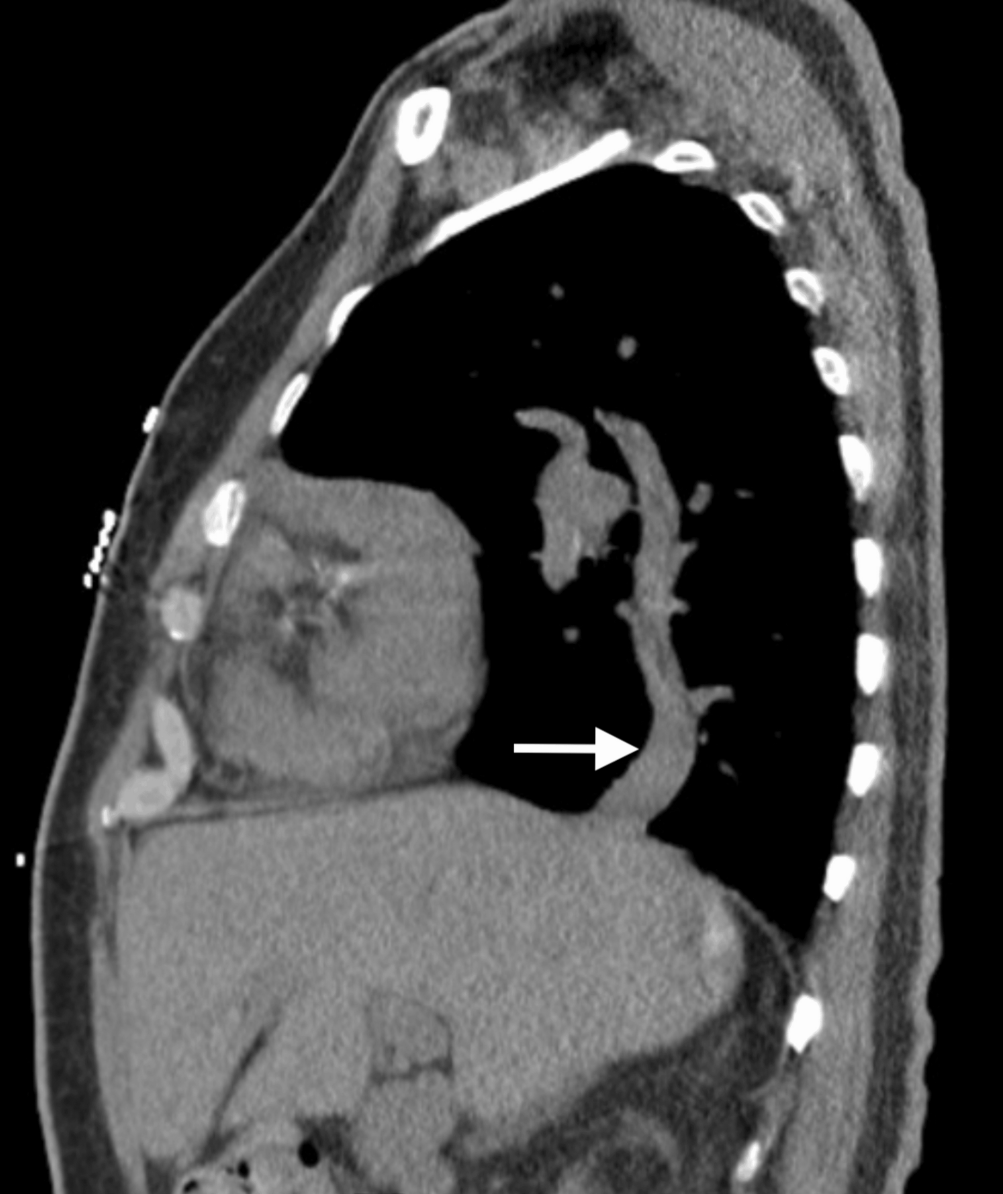
Chest computed tomography scan without contrast (sagittal reconstruction) demonstrating the scimitar vein, the aberrant connection of the pulmonary vein to the IVC

The electrocardiogram showed a sinus rhythm of 90 bpm, with no significant ST segment T wave changes (Figure 1). The patient did not present atrial tachyarrhythmias. The chest radiograph evidenced prominent vascularity bilaterally, including the right-sided scimitar vein (Figure 2). CT brain without contrast agent showed no evidence of acute intracranial hemorrhage, midline shift, or mass effect. Chest CT imaging without contrast agent demonstrated patchy ground glass mosaic pattern opacifications with right-sided predominance with an aberrant connection of the right lower pulmonary vein to the IVC, coronal view (Figure 3), and sagittal view (Figure 4). The ground glass mosaic pattern opacifications is a non-specific sign that is associated with different etiologies, including pulmonary edema, pneumonia or vascular abnormalities, such as scimitar vein. A few hours after the patient was initially evaluated, he developed altered mental status. Blood pressure at this time was 195/95mmHg, and heart rate was 86 bpm. The patient was admitted to the intensive care unit for hypertensive encephalopathy.

**Figure 5:**
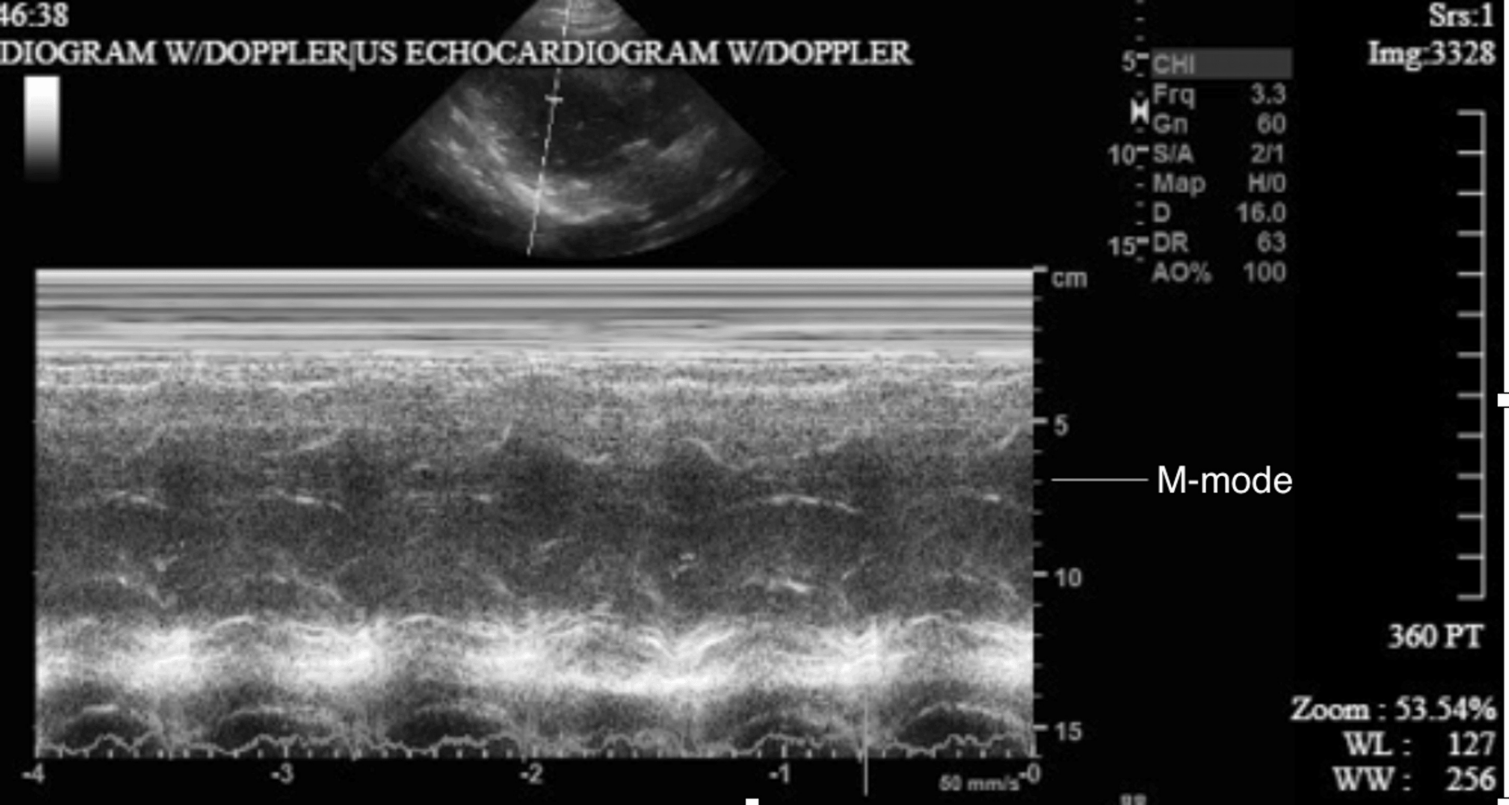
Transthoracic echocardiography showing preserved contractility on M-mode with left ventricular ejection fraction (LVEF) of 60-65%

The initial complete laboratory workup revealed hyperglycemia, 317 mg/dL (random), and negative troponin (<0.012). Urinalysis results include color brown, clarity cloudy, positive glucose, protein, leucocytes, blood, and few bacteria, negative for nitrite, leading to a urinary tract infection diagnosis hypothesis. Additionally, the patient presented dehydration, which is congruent with the elevated results from laboratory workup. The investigation continued and a transthoracic echocardiogram (TTE) (Figure 5) revealed a normal size left ventricle with preserved contractility (LVEF is 60-65%), moderate concentric left ventricular hypertrophy, and grade I diastolic dysfunction (impaired relaxation pattern). The right ventricle is reportedly of normal size and systolic function. Left and right atria were borderline dilated. No valvular abnormalities apart from mild mitral annular calcification, with noted mild mitral regurgitation and trace tricuspid regurgitation. In the subcostal view, a large, anomalous pulmonary vein (APV) drained into the IVC, with no evidence of pulmonary hypertension (mean pulmonary pressure < 25 mmHg). The patient did not have other associated congenital heart defects. He was diagnosed with incomplete scimitar syndrome. 

The patient's dehydration, hypertension, and urinary tract infection were treated. Once the blood pressure was controlled, the medication was switched to nifedipine 30 mg by mouth daily and labetalol 200 mg every eight hours with the diastolic pressure gradually being controlled, to avoid an ischemic event. He was started on empiric ceftriaxone 1 g IV once daily for the urinary tract infection. The stress response due to the infection may have contributed to the persistent hyperglycemia and the patient's decompensation. Hence, the patient showed clinical improvement with the resolution of symptoms and normalization of laboratory results, including the platelet count that gradually decreased.

With a multidisciplinary team evaluating the patient, the consensus was reached to continue with conservative management and regular outpatient follow-up with cardiology clinics. A surgical approach was not required due to the incomplete nature of the scimitar syndrome and normal mean pulmonary pressure as measured by echocardiography. He was discharged on nifedipine of 90 mg daily, labetalol of 300 mg every eight hours, clonidine of 0.2 mg twice daily, furosemide of 40 mg daily, metoprolol tartrate of 50 mg daily, and spironolactone of 25 mg daily and advised to follow up with a cardiologist as an outpatient.

## Discussion

Scimitar syndrome is a rare congenital heart defect with a prevalence of approximately two cases per million live births [4]. It is characterized as a PAPVR or TAPVR. The anomalous vein forms a curvilinear shape on imaging that resembles a scimitar Turkish sword, hence the name [6]. The severity of scimitar syndrome varies widely. It depends on the degree of left-to-right shunting generated by the anomalous vein and associated congenital heart defects [8]. In severe cases, symptoms tend to present early in infancy, and these patients often manifest heart failure, pulmonary hypertension (mean PAP of 25 mmHg or higher), or arrhythmias. However, patients with the incomplete form of the syndrome often remain asymptomatic. Thus, the presentation at older ages is rare. Frequently, the malformation is discovered incidentally on chest images. This may lead to underreporting of adult cases [9,10].

The underlying pathophysiology of scimitar syndrome and its long-term complications remain poorly understood [11], which highlights the need for further research and clinical data to guide clinicians in managing similar patients. We present a case of an adult diagnosed with incomplete scimitar syndrome with normal pulmonary pressure and no associated congenital heart defects. The diagnosis was made through chest CT and confirmed with TTE. He presented normal pulmonary pressure and no associated congenital heart defects. Of note, our patient had a preserved left ventricular (LV) ejection fraction (LVEF). LVEF is a surrogate of LV contractile function, and in the case of incomplete scimitar syndrome, it may remain preserved. The patient was treated for other comorbidities, and his condition improved under clinical management. Because of anatomical variances, technological restrictions, patient considerations, and the intricacy of incomplete scimitar syndrome, it might be difficult to obtain a clean subcostal image to visualize the condition. It is frequently easy to see the scimitar vein and obtain a good diagnosis using sequential imaging methods, which include CT.

Our case aligns with the findings of Dupuis et al. [12], who investigated hemodynamic characteristics in 122 adult patients with scimitar syndrome. The French study reported frequent associations with lung hypoplasia and vascular abnormalities in adults. However, it also noted that left-to-right shunt and pulmonary hypertension were less common, like our patient. Consequently, 85 out of 122 patients received medical management, with 92% showing favorable outcomes. These findings suggest that patients with normal pulmonary pressure and no congenital heart defect can have a good prognosis without the need for surgical intervention [12,13].

The presentation of incomplete scimitar syndrome can vary from asymptomatic to severe symptoms, such as pulmonary hypertension and arrhythmias in adult life (Table 2). Huang et al. [5] reported an asymptomatic adult with Scimitar syndrome with insignificant left-to-right shunt, negative for pulmonary hypertension or cardiac defects, who had excellent outcomes without surgery. It is important to highlight that, in certain scenarios, such as decompensated heart failure, cardiac defect associated, decompensated hypertension, severe infection, asthma crisis, or physiologic changes (e.g., pregnancy), as described by Althomali et al. [10], the condition of a previously asymptomatic adult may worsen, and timely diagnosis through imaging is essential [13,14].

**Table 2:** Literature review of case reports on adult incomplete scimitar syndrome Computed tomography (CT)

Author	Age/Sex	Symptoms	Qp/Qs	Pulmonary hypertension	Cardiac defects	Diagnostic testing	Management	Outcome/Follow-up
Huang et al. (2011) [5]	54 M	Asymptomatic	1.5	No, 18mmHg	None	CT scan, CT angiography	Conservative management	Stable, no complications
Althomali et al. (2017) [10]	33 F	Tachyarrhythmia	n/a	No	None	CT scan, CT angiography	Conservative management	Stable, no complications in 6 months
Saleh et al. (2017) [14]	70 F	Shortness of breath	3.6	Yes, 73 mmHg	None	CT scan	Conservative management	Stable, no complications
Colombo et al. (2014) [15]	38 F	Dyspnea, tachyarrhythmia	1.3	No, 24 mmHg	None	CT scan, CT angiography	Surgical repair	Obstruction of anastomosed vein. Repeated sternotomy. Improvement of symptoms

Saleh et al. [14] described an elderly female patient with recurrent chest pain and shortness of breath, who had severe pulmonary hypertension. Despite significant symptoms, the risks of surgery outweighed the benefits and the patient remained stable with conservative management. Therefore, we aim to underscore the importance of an individualized treatment approach, particularly in older adults and patients with significant comorbidities.

In addition, postoperative complications were reported by Colombo et al. [15] who presented a case of a young adult with significant progressive symptoms. The patient was diagnosed with incomplete scimitar syndrome without cardiac defect associated, low left-to-right shunt, and borderline pulmonary hypertension (24 mmHg), which has the potential to evolve over time, leading to severe symptoms and complications. For that reason, the patient reported by Colombo et al. [15] received surgical repair. However, her symptoms persisted. Obstruction of the anastomosed vein occurred and the patient underwent repair and reconstruction of the pulmonary vein. The case points out that thrombosis or stenosis of the scimitar vein is a serious complication [15].

The treatment for the syndrome in adults is currently controversial due to the rare occurrence of the disease, the complexity of a surgical repair, and the risk of stenosis [15]. Some authors recommend surgery intervention in cases marked by recurrent pulmonary infections, severe pulmonary hypertension, or heart failure [16]. Additionally, controlling pulmonary hypertension, optimizing hemodynamics and cardiac function, as well as providing sufficient postoperative analgesia, are all essential components of the effective perioperative care of complete or incomplete scimitar syndrome to ensure a positive patient outcome [17].

The risks associated with surgery must be carefully weighed. The recent study by Egbe et al. [18] suggests a 5-10% risk of post-surgical complications, such as venous stenosis, atrial fibrillation, or sinus node dysfunction. As a result, to minimize the risks, the current recommendations from American and European guidelines are to consider surgery for congenital heart disease in adults with PAPVR if a left-to-right shunt is higher or equal to 1.5 or functional impairment is present. Conservative management remains a reasonable approach, reducing unnecessary invasive procedures while preventing later complications [18-20].

## Conclusions

In summary, our case highlights a rare congenital malformation of incomplete scimitar syndrome and must not be overlooked as a benign condition. The adult presentation of the syndrome is often underrecognized, as patients remain asymptomatic. The presence of cardiac symptoms and pulmonary hypertension play a key role in long-term prognosis, management, and mortality. Surgical intervention is not recommended by current guidelines for asymptomatic patients with an insignificant left-to-right shunt. However, advanced imaging studies, such as computed tomography, echocardiography, and continuous clinical monitoring, are crucial for diagnosis, as in our case, and follow-up care, guiding management and improving the long-term outcome of this rare condition that may evolve with pulmonary hypertension and cardiac arrhythmias. The incomplete scimitar syndrome remains poorly understood due to the lack of clinical data. Further research is necessary to better comprehend the condition and raise awareness, optimizing management and long-term outcomes of adults with this condition.
